# Improved Porosity of Insect Proof Screens Enhances Quality Aspects of Zucchini Squash without Compromising the Yield

**DOI:** 10.3390/plants9101264

**Published:** 2020-09-24

**Authors:** Luigi Formisano, Antonio Pannico, Christophe El-Nakhel, Giuseppe Starace, Milena Poledica, Stefania De Pascale, Youssef Rouphael

**Affiliations:** 1 Department of Agricultural Sciences, University of Naples Federico II, 80055 Portici, Italy; luigi.formisano3@unina.it (L.F.); antonio.pannico@unina.it (A.P.); christophe.elnakhel@unina.it (C.E.-N.); youssef.rouphael@unina.it (Y.R.); 2 Department of Engineering for Innovation, University of Salento, 73100 Lecce, Italy; giuseppe.starace@unisalento.it; 3 Research and Development Lab, Sachim srl, 70017 Putignano, Italy; milena.poledica@sachim.it

**Keywords:** *Cucurbita pepo* L, anti-insect nets, mesh density, high tunnel, air temperature, airflow, qualitative parameters

## Abstract

In a global climate change environment, assuring optimal growing conditions is a difficult challenge, compromising the food supply for a rapidly rising population. The climatic conditions in the protected environment lead to high temperatures and fast insect development, impacting productivity and vegetables qualitative attributes. Consumers’ interest in healthy food requires sustainable tools to manage biotic and abiotic factors and, from this perspective, anti-insect nets represent an excellent “green” solution. For this purpose, our goal was to compare two different anti-insect nets on microclimate, production, and qualitative traits of *Cucurbita pepo* L. fresh fruits. The experiment was conducted in three separate polyethylene high tunnels, with 50 mesh anti-insect nets of different porosities being installed on the openings of two tunnels, while the third tunnel was a control without nets. Microclimate measurements, as well as yield, physiological, and phytochemicals variables, were assessed. The 50 mesh net led to a decrease in marketable yield (22.5%), fruit number (18.0%), CO_2_ net assimilation rate (6.0%), and transpiration rate (29.5%). Total soluble solids, antioxidant activities and total ascorbic acid concentration had an opposite trend. The 50 mesh AirPlus net improved quality aspects of zucchini fruits by increasing total ascorbic acid, total phenols, and antioxidant compounds, with no negative impact on yield.

## 1. Introduction

Recent climate changes are severely affecting agriculture and endangering food supply for future generations [1], especially in countries with lower socioeconomic resources and a higher risk of poverty, thus introducing new challenges for food production. The agricultural sector supplies about 50% of the nourishment needed by the world increasing population, which is expected to reach 9.7 billion in the coming decades [2]. However, the effective yield achieved is only 50% [3] of what would be potentially obtainable, due to biotic and abiotic factors undermining agricultural production, notably insects and photothermal stress [4]. In a global climate change environment, assuring optimal growing conditions is an arduous challenge, especially in warm Mediterranean areas where high temperatures, water, and insects’ proliferation are limiting factors, reducing productivity and qualitative vegetable traits [5].

The ever-increasing interest of consumers in healthful food has led to a “consumer-oriented” revolution and new quality perceptions. Quality is no longer relegated exclusively to food appearance but also includes its bioactive phytochemical content, combining healthfulness with gastronomic pleasure [6]. The consumer ascribes to food a supporting role for human wellness due to the content of beneficial bioactive compounds. Accordingly, the market is tailored to the requests of an increasingly informed and health-conscious consumer, orienting growers towards high quality and eco-sustainable production. On the other hand, it is well established that the climatic conditions in high tunnels may facilitate the rapid development of pests [7], requiring eco-friendly tools for their containment [8] and physical barriers represent an excellent “green” solution.

The exclusion performance of anti-insect nets depends on geometrical and structural hole patterns [9], where an appropriate net selection can achieve up to 90% control of a designated pest [10]. Today, the manufacturers have the knowledge to develop and produce various kinds of agro-textiles with different mechanical, physical, and radiometric features, satisfying the specific requirements of growers [11]. Aside from the aspects outlined above, most anti-insect nets for agricultural use are manufactured by a weaving process. Several vertical and horizontal warp and weft plastic threads are overlapped and woven, making a regular structure variable in size, according to the insect to be excluded and with a specific porosity (percentage of the ratio between open net area and total net area) [11]. From a commercial viewpoint, anti-insect nets are classified by mesh number, describing the number of openings per linear inch [12]. The performance of insect exclusion screens is founded on avoiding insect thorax passage through net mesh (“prison effect”) [13] and, theoretically, small hole nets are more effective. However, the lower porosity of fine mesh nets, leads to a high static pressure drop [14,15], resulting in inadequate air exchange and reduced ventilation [16], hence exposing crops to abiotic stress that affects crop growth and production, while representing a barrier for pollinators [17]. Consequently, a high differential in temperature and relative humidity occurs between the indoor and outdoor growing environment [18,19].

It is well acknowledged that high temperatures induce morpho-physiological, molecular, and biochemical modifications in plants [4,20] as an adaptive response to heat stress. High temperatures interfere with primary metabolism (photosynthetic and respiratory processes) [21], inhibit bud and root growth, stimulate leaf abscission, impair fruit set, damage fruits [22], and decrease root assimilation efficiency [23]. Additionally, heat stress alters enzymatic activity, modifies chloroplast proteins [24,25] and enhances soluble sugar accumulation [26,27,28]. Furthermore, heat promotes the production of high quantities of reactive oxygen species (ROS), resulting in a lower biosynthesis of photosynthetic pigments [29,30,31] which reduces the activity of Photosystem II [32]. Blooming and fruit set are similarly sensitive to high temperature stress, as evidenced by studies on zucchini squash [33,34] and tomato [32,35]. Not lastly, high temperatures influence secondary metabolism by stimulating biosynthesis and the accumulation of antioxidant compounds as observed in watermelon and tomato [36].

Zucchini (*Cucurbita pepo* L.) is a vegetable that is gaining popularity in Europe, representing a resource for the horticultural chain, ranking fourth among retail vegetables. Among the European countries, Italy has a greenhouse production of 218,950 tons [37] and an annual per capita consumption of 9 kg [38]. Moreover, the increased consumer demand in the national and international market for fresh fruits available all-year-round has made zucchini greenhouse cultivation increasingly popular. Based on our knowledge and the examined literature, there is an evident lack of research assessing the impacts of the microclimate induced by anti-insect nets on the production and quality attributes of zucchini squash. Recent and interesting comparable studies on cucumber have highlighted the positive effects induced by insect exclusion screens in containing cucumber beetles (*Acalymma vittatum* Fabr.) in high tunnels while ensuring adequate ventilation [39]. Undoubtedly, the few contributions available were mainly focused on evaluating the effects of insect exclusion on crops yield with no emphasis on the interaction between the microclimate and the qualitative and quantitative vegetables response.

Based on the considerations mentioned above, the presented research was aimed at assessing the influence of the microclimate change induced by two 50 mesh anti-insect nets with different porosity on the qualitative and quantitative aspects of zucchini fruits. As far as we know, this is the first research investigating these aspects, establishing a basis for future studies.

## 2. Results

### 2.1. Microclimate Parameters

**Figure 1:**
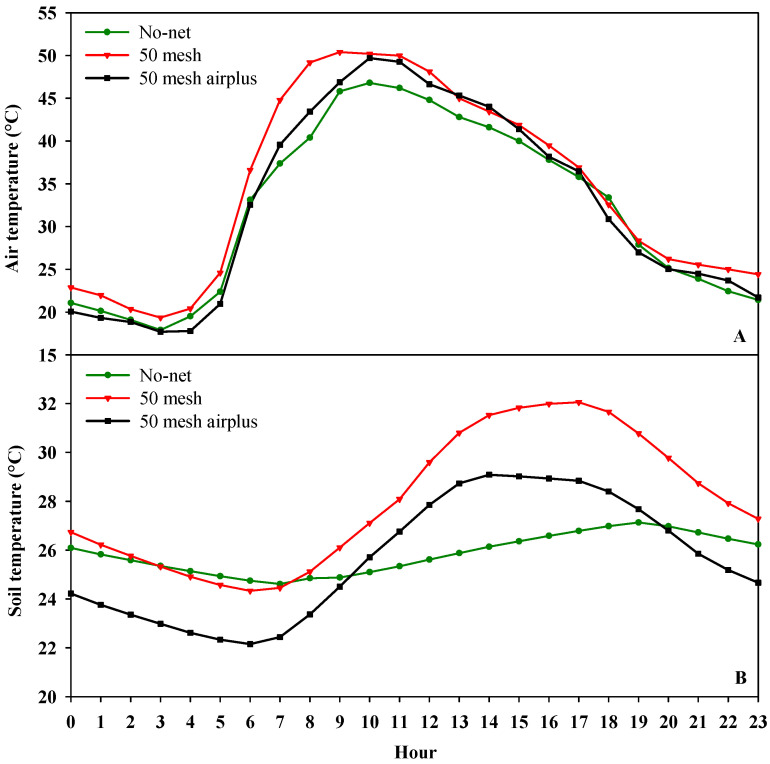
Hourly air temperature (**A**) and soil temperature (**B**) recorded inside the high tunnels covered with nets and without nets.

**Figure 2:**
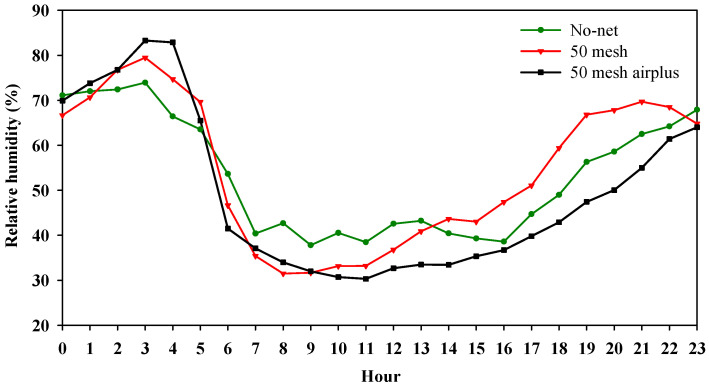
Hourly air relative humidity recorded inside the high tunnels covered with nets and without nets.

**Figure 3:**
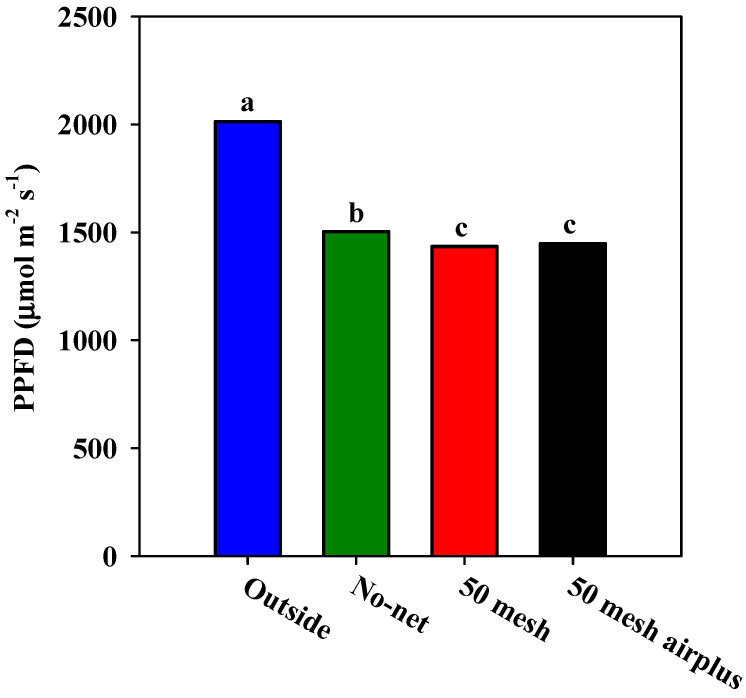
Average PPFD (Photosynthetic Photon Flux Density) values recorded inside the high tunnels covered with nets and without nets and outside the high tunnels.

Figure 1A,B and Figure 2 show, respectively, the hourly air temperature, the hourly soil temperature and relative humidity inside the high tunnels. As regards air temperature, both nets recorded higher values when compared to the control, especially during the warmer part of the day (from 10:00 to 15:00). However, during the early hours of the day (from 6:00 to 10:00), the 50 mesh AirPlus net showed an average lower air temperature (−10%) than 50 mesh net (Figure 1A). Similarly, the soil temperature throughout the day was lower in the high tunnels covered with 50 mesh AirPlus net compared to the 50 mesh one (Figure 1B). In particular, from 8:00 to 20:00, it observed an increase of 5 and 14% of the soil temperature under the 50 mesh AirPlus net and the 50 mesh, respectively, compared to the unscreened control (Figure 1B). The daily trend of the relative air humidity showed, from 10:00 to 23:00, that the 50 mesh AirPlus net recorded a lower value than both the 50 mesh net and the control (Figure 2). The greenhouse cover film affected the PPFD (photosynthetic photon flux density) resulting in an average reduction of 30% compared to the outside (Figure 3). Of note, the use of the nets reduced light radiation by only 5% compared to the control without nets.

### 2.2. Influence of Anti-Insect Nets on Yield and Yield Components

**Figure 4:**
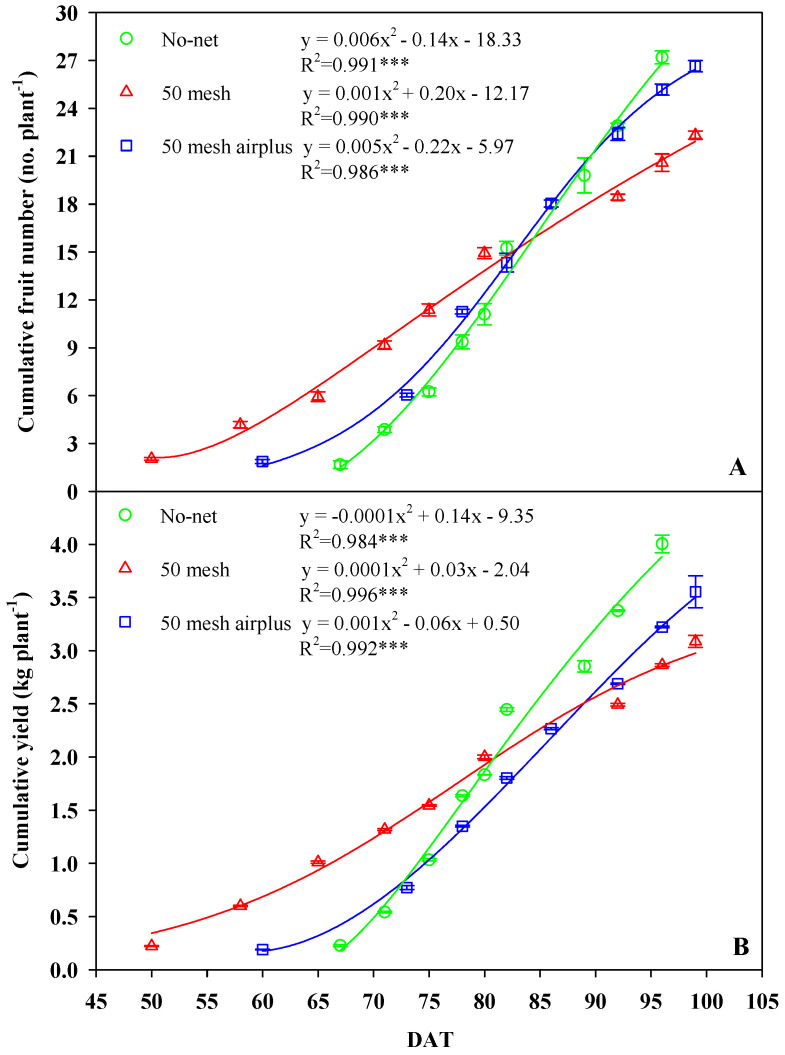
Effects of anti-insect nets on the cumulative number of fruits (**A**) and yield (**B**) per plant at different days after transplant (DAT).

The yield and yield components of zucchini squash produced under the different anti-insect nets are presented in Table 1 and Figure 4. The yield and the number of fruits per plant were influenced by treatments, whereas the mean weight of the fruits showed no significant difference (Table 1). In particular, the yield and number of fruits grown in the 50 mesh net-covered high tunnel decreased by 23% and 18%, respectively, in comparison to unscreened control. Interestingly, the 50 mesh anti-insect net resulted in an earlier production of 10 and 17-days, compared to the 50 mesh AirPlus net and control, respectively (Figure 4A,B). Furthermore, up to 80 days after transplant, the 50 mesh net resulted in improved productivity, regarding both yield and number of fruits, while an opposite trend was observed in the following days until the end of the cycle. In fact, after 80 DAT (days after transplant) lower production was evident in 50 mesh nets-treated plants compared to AirPlus 50 mesh net and control (Figure 4A,B).

### 2.3. Influence of Anti-Insect nets on Physiological and Biochemical Parameters

With the exception of soil plant analysis development (SPAD) index, all the analyzed physiological parameters showed significant differences between the different treatments. The leaf net CO_2_ assimilation rate (A_CO2_), transpiration (E), and maximum quantum efficiency of PSII photochemistry (F_v_/F_m_) showed a significant decrease in plants treated with 50 mesh net, compared to the 50 mesh AirPlus net and control. Specifically, in plants covered by 50 mesh nets, average values of A_CO2_, E, and F_v_/F_m_ were, respectively, 5.77, 29.6, and 6.76% lower than those recorded in the control (Table 2). An opposite trend was observed for stomatal resistance (r_s_) and intrinsic water use efficiency (WUEi) that recorded the highest values in the 50 mesh treatment (Table 2).

### 2.4. Fruit Juice pH, Total Soluble Solids, and Dry Matter

Total soluble solids (TSS) and dry matter (DM) of the fruits showed significant differences among the treatments (Table 3), while no difference was found for the fruit juice pH (6.35, on average). The total soluble solids showed an increment of 47.9% in fruits grown under 50 mesh net with respect to the control (Table 3). Similarly, both nets resulted in a significant increment in DM content of the fruits (+19.7%, on average) compared to untreated control (Table 3).

### 2.5. Analysis of Total Ascorbic Acid, Total Phenols, and Antioxidants Activities

The anti-insect nets significantly affected the total ascorbic acid content, total phenols content and the antioxidant activities (Table 3). In particular, hydrophilic antioxidant activity (HAA) and ABTS antioxidant activity of fresh zucchini fruits ranged from 9.93 to 10.58 mmol ascorbic acid eq. 100 g^−1^ dw and from 17.4 to 23.1 mmol Trolox eq. 100 g^−1^ dw, respectively. Both antioxidant activities were significantly higher in the fruits of plants grown under nets. Similarly, the total ascorbic acid content in the fruits of nets-protected plants was on average 9.7% higher than that recorded in the untreated control (Table 3). In contrast, the total phenols content increased by 18.9% only in plants grown under 50 mesh AirPlus compared to control.

## 3. Discussion

Anti-insect nets are a sustainable and efficient approach for insect exclusion in protected environments [8]. However, small hole nets lead to detrimental increased temperature and relative humidity [40,41]. The aim of our research was the assessment of anti-insect nets with different porosity on the induced microclimate and on the productive and qualitative performance of zucchini squash plants. In the cultivation area where the experiment was conducted, due to the distinct climatic conditions (warm spring–summer and constant wind), zucchini plants were particularly vulnerable to early attacks by insects and pathogenic fungi. Therefore, especially in early growth stages, when the plants are particularly susceptible, phytopathogenic adversities can quickly lead to the death of young and still poorly lignified plants. For this reason, fungicide treatments (one with penconazole and two more with wettable sulfur) were carried out in all the tunnels, at the same time and the same dosage, to eliminate any variability resulting from their use. Likewise, at the beginning of the test (0 DAT), in order to eliminate any wintering insects, a selective insecticide treatment based on pirimicarb was carried out in all the tunnels (screened and unscreened) by foliar spray application. Subsequently, it was decided to carry out careful monitoring of the biotic pressure through the use of chromotropic traps placed in all the tunnels, intervening with insecticide treatments when the intervention threshold was exceeded. In this regard, in the screened tunnels, even if a certain biotic pressure was present, the intervention thresholds never exceeded during the entire crop cycle; in contrast in the unscreened tunnel, five insecticide treatments with potassium salts of fatty acids C14-18 were necessary to maintain the biotic charge at levels comparable to the screened tunnels, and thus neutralize any variability caused by the different grade of insect attacks. This finding confirms inter alia the effectiveness of anti-insect nets in controlling biotic pressure in the present experiment. The potassium salts of fatty acids are readily degraded via photochemical processes without leaving residues on the vegetation [42,43]; in contrast, they are selectively active on target pests by dissolving the waxes present in the insect cuticle causing their death by dehydration [44,45].

Our findings demonstrated an evident influence of nets on inner microclimate. The higher air and soil temperature and relative air humidity recorded are in agreement with previous comparable studies [19,46]. It is noteworthy that the lower temperature of air and soil, as well as relative air humidity, were recorded using the 50 mesh AirPlus net. The improved performance of the 50 mesh AirPlus net is due to the employment of a thinner high density polyethylene (HDPE) filament (Arrigoni Harlene HT^®^, Uggiate Trevano (CO), Italy) resulting in a higher porosity at the same mesh number. Teitel and Shklyar [14] demonstrated that increased porosity is associated with reduced static pressure drop, and thus an improvement in airflow and microclimatic parameters. Both nets significantly reduced light transmission compared to the unscreened control; however, this decrease in PPFD around 60 μmol m^−2^ s^−1^ did not actually affect plant growth. Nevertheless, agreeing with Klose and Tantau [47], the lower spacing between adjacent threads does not necessarily imply lower light transmission; probably, soil dust accumulated on the nets and the structure of the threads, were involved in masking light radiation and hence reducing PPFD. The AirPlus 50 mesh net showed a yield and number of fruits in range with the typical greenhouse production of zucchini squash [48], in contrast the 50 mesh net recorded values below the reference standards. Indeed, the heat stress caused by lower porosity of 50 mesh net affected the physiological activities of zucchini plants, reducing the yield and the number of fruits. In support of our data, different investigations on tomatoes reported adverse effects of heat stress on radical conductance [49], source and sink activities [50], and carbon transport to the vegetative apex [51]. However, low fruit number is probably due to fertilization and embryo development defects under high-temperature conditions, as found in previous works on tomato [32,35] and zucchini [33]. Other studies on zucchini revealed that high temperatures are also related to the production of immature and “attached-flowers” fruits, leading to a reduced yield [34]. In fact, at the beginning of the growing cycle, due to the lower recorded soil and air temperatures, anti-insect nets positively influenced plant growth by rising the temperature to an average, convenient for early production. As the growing period advanced (June/July), the opposite trend occurred as temperatures rose and caused adverse conditions to zucchini production. Heat stress caused flowers and fruit drop, leading to a lower total yield, mainly in plants grown using the 50 mesh net.

The anti-insect nets also affected photosynthesis and transpiration. Notably, the 50 mesh net caused lower net CO_2_ assimilation (A_CO2_) and maximum quantum efficiency of Photosystem II (F_v_/F_m_), attributable to a reduced biosynthesis of photosynthetic pigments and photosystem II activity or both effects combination. High temperatures altered the permeability and structure of cell membranes and reduced the activity of several enzymes [25] and the regenerative ability of 1.5-bisphosphate ribulose carboxylase (RuBisCo) [49,52], leading to a reduced carbon fixation, and thus affecting the adjustment capacity of the photosystem II [53]. Furthermore, heat stress impaired and disrupted the oxygen-evolving complex [54], resulting in the production of potentially harmful reactive oxygen species [29,30], affecting the biosynthesis of chlorophyll pigments and reducing the photosynthesis. Moreover, Tewari and Tripathy [55] demonstrated that under high-temperature conditions, chlorophyll biosynthesis in *Cucumis melo* L. plants was reduced by 60% due to the deactivation of the 5-aminolevulinate dehydratase enzyme involved in pyrrole biosynthesis. Similarly in tomato, Camejo et al. [32] reported a reduction in chlorophyll/carotenoid ratio content.

Additionally, transpiration is the principal leaf cooling system, and the stomata play a fundamental role in its regulation, offering a low resistance way for gas diffusion through the leaf. Under optimal water and high sunlight levels, the leaf’s demand for CO_2_ is highest, and thus stomatal resistance reduces while the transpiration rate increases. However, at high-temperature levels, this process is impaired, as occurred in 50 mesh net treatment. The lower CO_2_ request, as a result of damaged photosynthetic apparatus and the reduced biosynthesis of photosynthetic pigments, resulted in a decrease in the transpiration rate and an increase in the stomatal resistance and intrinsic water use efficiency (WUEi), through which the plants attempted to minimize water loss by closing the stomata and decreasing transpiration. Further explanations were provided by Taiz et al. [56] and, probably, the reduced transpiration derives from low internal airflow, leading to higher resistance of the air boundary layer at the leaf surface, or from the accumulation of ABA (abscisic acid) in the leaves in response to high temperatures.

Plants in addition to synthesizing primary compounds as proteins, lipids, carbohydrates, and acids, produce a wide range of secondary metabolites indirectly involved in growth and development, as well as relevant defensive properties [56]. Scientific studies [57,58] highlighted that plants’ phenolic compounds protect human cells during the first stages of cancer development and exhibit an elevated antioxidant activity that exerts beneficial actions on vascular and nervous systems [59], mitigating the side effects of certain diseases including dementia, Alzheimer’s and Parkinson’s [60,61]. Phenolic compounds contribute to an increase in quality of vegetables, which is related to intrinsic (genotype) and extrinsic (environment) factors [62]. Zucchini squash fruits have a high water and macronutrients content as well as a low protein and fat content. Additionally, they have a high content of hydrophilic (vitamin C, niacin, vitamin B-6, riboflavin, and thiamine) and lipophilic (vitamin E, β-carotene, vitamin A, and vitamin K) antioxidant compounds [63]. Many studies have pointed out a positive quality change induced by heat stress, attributable to increased antioxidants compounds as a defensive response to ROSs accumulation [23,31]. According to our experiment, both anti-insect nets induced an increase of TAA, compared to USDA [63] values, and an increase of antioxidant activities. In contrast, the total phenols content was significantly higher in fruits cultivated under the 50 mesh AirPlus net treatment. Investigations on watermelon and tomato revealed that antioxidant molecules produced at high temperatures represent a mechanism of resistance to heat stress [36]; meanwhile, Wahid et al. [23] suggested that they might provide an additional control function of the leaf’s osmotic potential to reduce water loss through transpiration, which is supported by our results. However, the lower accumulation of the total phenols occurring in the 50 mesh net treatment could be the result of plants being unable to adapt rapidly to the high thermal stress, leading to an inhibition of phenolics biosynthesis.

Moreover, anti-insect nets also influenced both TSS and DM contents of fruits. As for TSS, compared with the 50 mesh treatment, higher amounts were recorded in fruits, reflecting the higher heat stress induced by the net, making our results aligned with different researches. Indeed, some studies evidenced an increased production of primary metabolites like proline, glycine betaine, and especially of soluble solids in plants exposed to heat stress [23], in order to improve the protein and cellular membrane stability and to regulate the osmotic potential, representing an indicator of thermal stress. Carbohydrates such as sucrose, the main photosynthesis product, regulate plant development and allow carbon allocation and sugar signaling, as suggested by Roitsch and Gonzalez [26]. Furthermore, an antioxidant action of sugars [28] and ROS scavenger function [27] was shown.

## 4. Materials and Methods

### 4.1. Growth Conditions, Treatments and Experimental Design

**Figure 5:**
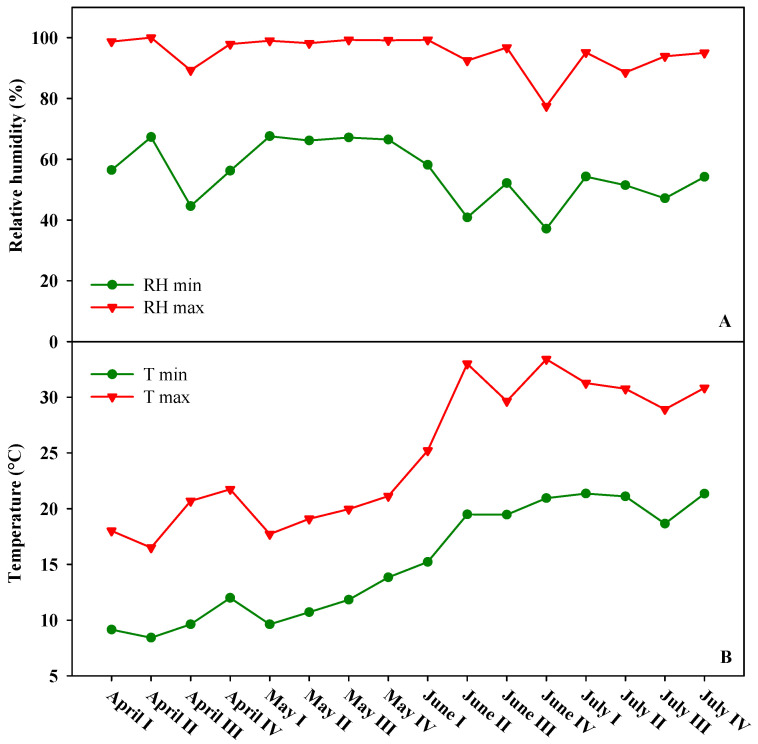
Minimum and maximum relative air humidity (**A**) and air temperature (**B**) recorded outside the high tunnels during the growing season at the experimental site.

The present experiment was carried out in 2019 growing season at the greenhouse complex at the experimental farm “Torre Lama” of the University of Naples, situated in Bellizzi (Salerno, southern Italy; latitude 43°31′ N, longitude 14°58′ E, altitude 60 m). The main physical and chemical soil characteristics at the experimental site were clay loam texture (46% sand, 24% silt, and 30% clay), electrical conductivity (EC): 0.16 dS m^−1^, pH: 7.7, total nitrogen (N): 0.11%, and organic matter: 1.21% (*w*/*w*). The Olsen phosphorus and exchangeable potassium were 88 and 980 mg kg^−1^, respectively. The quality of the irrigation water was characterized by high bicarbonate content. The concentrations of ions expressed as mg L^−1^ were calcium (86); chloride (9); magnesium (20); sodium (7); potassium (und.); sulfate (9); nitrate (4.5); and bicarbonate (285). The values of pH and EC were 7.5 and 0.43 dS m^−1^, respectively. Water was provided by a drip irrigation system consisting of a main polyethylene pipeline (32 mm diameter and 2 atm operating pressure) with a series of semi-compensating dripping wings (16 mm diameter and 60 cm interpolation). The growing system was made of three single high tunnels 30 m long, 7.2 m wide, and 2.8 and 4.5 m high at the eaves and ridges, respectively, each covered with a polyethylene film applied to the greenhouse gables, roof, and the lower part of the side walls (up to a height of 0.6 m above the ground). The high tunnels were irradiated by natural sunlight while relative humidity and temperature were managed through natural ventilation. Figure 5 show minimum and maximum relative air humidity and air temperature recorded outside the high tunnels during the growing season at the experimental site.

Seeds of parthenocarpic zucchini squash (*Cucurbita pepo* L.), variety Zufolo F1 (Olter, Piacenza, Italy) were germinated in vermiculite on 14 March 2019. Seedlings were transplanted on 1 April, at the two true-leaf stages in three single rows with a plant distance of 1.6 and 0.6 m inter- and intra-rows, respectively, giving a density of 1 plant m^−2^.

At transplant, a foliar spray insecticide treatment with pirimicarb at the dose of 2.3 g/L (Pirimor 17.5, Adama, Grassobbio (BG), Italy) was carried out inside the tunnels to eliminate any wintering insects. During the experiment, additional five insecticide foliar spray treatments with potassium salts of fatty acids C14–18 (soft soap) at the dose of 15 mL/L (Acaridoil 13 SL, Agrowin Biosciences, Bergamo, Italy) were applied inside the unscreened tunnel when the intervention threshold (number of insect/trap) was reached by monitoring the insects’ count through chromotropic traps. The last insecticide treatment was carried out at 78 DAT. Moreover, at 25 DAT powdery mildew (*Sphaerotheca fuliginea*) protection was performed inside all the tunnels with penconazole foliar spray treatment at the dose of 0.5 mL/L (Topas 10 EC, Syngenta, Milano, Italy). Subsequently two foliar spray treatments with wettable sulfur at the dose of 1.5 g/L (Wettable Sulfur, Bayer, Milano, Italy) were carried out.

**Figure 6:**
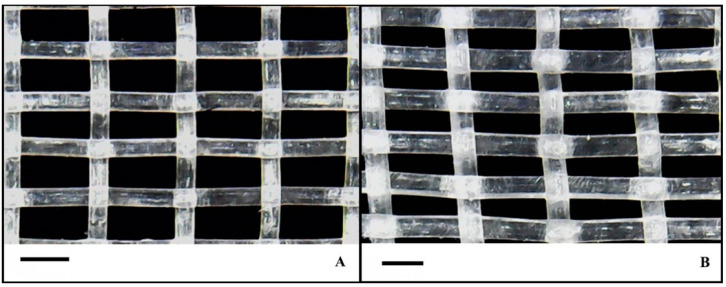
Arrigoni Biorete^®^ 50 Mesh AirPlus with Harlene HT^®^ HDPE (high density polyethylene) monofilament (**A**) and Arrigoni Biorete^®^ 50 Mesh with standard HDPE monofilament (**B**). Scale bars are 0.5 mm in (**A**,**B**).

The experimental treatments consisted of two 50 mesh size anti-insect nets differing in porosity and permeability to air, and an unscreened control treatment. The study was conducted to compare the influence of the two anti-insect nets that covered the sidewalls and ventilation openings of the two tunnels, whereas the third tunnel was used as a control (unscreened). The anti-insect nets features were as follows: (1) Biorete^®^ 50 mesh (Arrigoni S.p.A, Uggiate Trevano, Italy; Ø warp-weft: 0.23/0.23; warps-wefts per cm: 20/10; hole dimension: 0.27 × 0.79 mm; permeability to air: 36%; ventilation reduction: 32%; shade factor: 13); (2) Biorete^®^ 50 mesh AirPlus (Arrigoni S.p.A, Uggiate Trevano, Italy; Ø warp-weft: 0.17/0.17; warps-wefts per cm: 20/11.7; hole dimension: 0.33 × 0.68 mm; permeability to air: 47%; ventilation reduction: 30%; shade factor: 11). The improved air permeability was achieved by using UV-stabilized high density polyethylene (HDPE) monofilament (Arrigoni Harlene HT^®^) that resulted in thinner and more resistant net and leading to an increased hole size for the same mesh number (Figure 6). Treatments were arranged in a completely randomized design where the three treatments were arranged in a cross section within the three tunnels to remove the variation due to the uneven conditions (experimental error) across tunnels. In particular, each horizontal strip that covers the three tunnels was a block of the completely randomized design that contains all three treatments. A total of 150 plants were transplanted in each high tunnel (50 plants for each tunnel cross section).

### 4.2. Microclimate Measurements

Two WatchDog A150 data loggers (Spectrum Technologies Inc., Aurora, IL, USA; ±0.6 °C/±3% Temp/RH accuracy) separated by 10 m, were located in the midpoint of each high tunnel and placed at a height of 0.5 m above ground level, to record air temperature and relative humidity. The soil temperature was recorded by negative temperature coefficient (NTC) HI141BH thermo logger with external sensor (Hanna instruments^®^, Woonsocket, RI, USA; ±0.5 °C accuracy) placed in the middle of each high tunnel at a depth of 6 cm. Outside climatic data were measured using a meteorological station Davis Pro2^TM^ Plus Stations 6163 (Davis Instruments, Hayward, CA, USA), located 20 m away from the high tunnels. All external sensors were placed at a height of 7 m above ground level. The climatic data were collected at an interval of 30 min. Mean temperatures from April to July (2015–2018) were presented in Figure S1, in order to show the redundancy of the mean temperatures among the recent four years during the same period and same zone of our experimental site. These data were collected by the meteorological station of Battipaglia (Salerno, Italy).

Twenty PPFD (photosynthetic photon flux density) measurements were recorded between 11:00 and 13:00 h, inside and outside the high tunnels, using a handheld spectral radiometer (MSC15, Gigahertz-Optik, Turkenfeld, Germany) at 0, 50, and 99 days after transplant.

### 4.3. Yield and Fruit Quality Measurements

The experimental trial was conducted from 30 May to 17 July 2019. The fruits of six plants per plot were harvested three times per week when they reached 12 cm in length (marketable fruits). For each plant, right after harvesting, the fresh weight and number of the fruits were recorded. Deformed or undersized fruits were considered unmarketable. 

After 102 DAT, eight representative fruits per plot, free of disease symptoms or visible defects, were sampled and analyzed for quality parameters. The mesocarp of each fruit was homogenized in a Waring^®^ blender (2 L capacity; Model HGB140, McConnellsburg, PA, USA) for 1 min and then filtered. The extracted juice was measured by a digital refractometer Atago N1 (Atago Co. Ltd., Tokyo, Japan) to determine the total soluble solids (TSS) content expressed as °Brix at 20 °C. A pH meter (HI-9023; Hanna instruments^®^, Woonsocket, RI, USA) was used for determining the fruit juice pH. One hundred grams of the fruit juice was dried in a forced-air oven at 80 °C for 72 h until reaching constant weight, for dry matter (DM) percentage determination. Sections of sampled fruits were immediately placed in liquid nitrogen and then stored at −80 °C for further qualitative analysis.

### 4.4. Soil Plant Analysis Development Index (SPAD), Leaf Gas Exchange, and Chlorophyll Fluorescence

At 99 DAT, measurements of the SPAD index were performed on fully expanded leaves of six plants per plot using a portable chlorophyll meter SPAD-502 (Minolta Corp. Ltd., Osaka, Japan). A single average SPAD value for each replicate was obtained by measuring twenty leaves randomly.

On the same date, measurements of gas exchange and fluorescence emission were conducted between 11:00 and 13:00 h on the youngest fully expanded leaves. A portable gas exchange analyzer (LCA-4; ADC BioScientific Ltd., Hoddesdon, UK) equipped with a broadleaf chamber was used to determine the net CO_2_ assimilation rate (A_CO2_), stomatal resistance (r_s_), and transpiration (E). PPFD, relative humidity (RH), and CO_2_ concentrations were set at ambient values (700 ± 50 μmol m^−2^ s^−1^, RH 55 ± 5%, and 365 ± 5 ppm, respectively) and the flow rate of air was 400 mL s^−1^. Intrinsic water use efficiency (WUEi) was calculated as A_CO2_/E ratio.

Modulated chlorophyll fluorescence was performed on six plants per plot on dark-adapted (for at least 10 min) leaves, using a portable fluorometer F_v_/F_m_ Meter (Opti-Sciences Inc., Hudson, NH, USA). The ground fluorescence signal, F_o_, was induced on 10′ dark-adapted leaves, by a blu LED internal light of 1–2 μmol m^−2^ s^−1^. The maximal fluorescence intensity in the dark-adapted state (F_m_) was induced by a 1s saturating light pulse of 3000 μmol m^−2^ s^−1^. The maximum quantum efficiency of open Photosystem II (PSII), Fv/Fm, was calculated as (F_m_-F_o_)/F_m_, according to Kitajima and Butler [64]. 

### 4.5. Analysis of Total Ascorbic Acid, Total Phenols, and Antioxidants Activities

Total ascorbic acid (TAA) was assessed by spectrophotometric detection of fresh fruit material as described by Kampfenkel et al. [65]. TAA was measured by UV–VIS spectrophotometry (Hach DR 4000; Hach Co., Loveland, CO, USA). The solution absorbance was measured at 525 nm.

The Folin–Ciocalteu procedure [66] was used for evaluating the total phenolic content. A sample of lyophilized material was extracted in 60% methanol/water (*w*/*v*) with gallic acid as standard. UV–VIS spectrophotometer was used to measure the absorbance at 765 nm.

Two hundred milligrams of lyophilized zucchini fruits underwent different extraction procedures in order to quantify the antioxidant activity. The hydrophilic fraction (HAA) was measured as described by Fogliano et al. [67]. Whereas, a wider fraction of the antioxidant activity (ABTS AA) was measured by the method of Pellegrini et al. [68], where 2,2’-azinobis (3-ethylbenzothiazoline-6-sulfonic acid) (ABTS) radical cation decolorization assay was used. The absorbance of HAA and ABTS AA solutions were measured at 505 and 734 nm, respectively, by UV–Vis spectrophotometry.

### 4.6. Statistical Analysis

All experimental data were analyzed by ANalysis Of VAriance (ANOVA) using the software package SPSS 10 for Windows, 2001 (SPSS Inc., Chicago, IL, USA). After the verification of the normality through the test of Shapiro–Wilk, Duncan’s Multiple Range Test (DMRT) was performed at *p* ≤ 0.05 on each of the significant measured variables.

## 5. Conclusions

The increasing consumer attention towards healthy foods has driven growers to research alternative eco-sustainable agronomic practices to chemical insecticides. In this perspective, anti-insect nets represent a valid eco-friendly solution. Although small hole nets are more effective, their high resistance to airflow reduces ventilation, resulting in a detrimental increase in temperature and humidity, representing a critical issue in the warm Mediterranean region. The results obtained suggest that the different porosity of tested insect nets modulates the yield and its precocity as well as the quality aspects of zucchini fruits. Particularly, the 50 mesh AirPlus resulted in an overall improved qualitative and physiological parameter without any reduction in yield and number of fruits. Its intrinsic highest porosity led to a better air exchange rate, resulting in an increased dry matter, antioxidant activities, total phenols, total ascorbic acid, CO_2_ assimilation rate, and transpiration. Based on the results obtained, it appears promising to evaluate the effects of 50 mesh AirPlus on yield and quality attributes of other greenhouse fruit vegetables. However, the 50 mesh net could be an excellent tool for growers to achieve earlier production in autumn-winter or late-winter crop cycles when higher temperatures are appreciated.

## Figures and Tables

**Table 1:** Effects of anti-insect nets on yield, number of fruits per plant, and mean fruit weight of zucchini squash.

Treatments	Yield	Fruit Number	Mean Weight
	(kg fw Plant^−1^)	(no. Plant^−1^)	(g fw Fruit^−1^)
No-net	4.00 ± 0.17 a	27.20 ± 0.41 a	147.10 ± 4.25
50 mesh	3.09 ± 0.06 b	22.32 ± 0.26 b	138.40 ± 4.05
50 mesh AirPlus	3.55 ± 0.18 ab	26.64 ± 0.35 a	133.40 ± 6.56
Significance	*	***	ns

Means within each column followed by different letters are significantly different (*p* ≤ 0.05) according to Duncan’s multiple range test. ns, *, *** Non-significant or significant at *p* ≤ 0.05, and 0.001, respectively. All data are expressed as mean ± standard error, n = 3.

**Table 2:** Effects of anti-insect nets on Soil Plant Analysis Development Index (SPAD index), net photosynthesis (Aco_2_), stomatal resistance (r_s_), transpiration (E), intrinsic water use efficiency (WUEi), and chlorophyll fluorescence of zucchini squash.

Treatment	SPAD Index	A_CO2_	r_s_	E	WUEi	Fluorescence
		(μmol CO_2_ m^−2^ s^−1^)	(m^2^ s^1^ mol^−1^)	(mol H_2_O m^−2^ s^−1^)	(μmol CO_2_ mol^−1^ H_2_O)	Fv/Fm Ratio
No-net	47.19 ± 0.59	12.31 ± 0.16 a	6.65 ± 0.15 b	3.01 ± 0.29 a	4.23 ± 0.28 b	0.74 ± 0.00 a
50 mesh	45.62 ± 0.61	11.60 ± 0.15 b	7.40 ± 0.32 a	2.12 ± 0.26 b	5.83 ± 0.62 a	0.69 ± 0.02 b
50 mesh AirPlus	46.16 ± 0.73	12.26 ± 0.13 a	6.13 ± 0.18 b	3.81 ± 0.29 a	3.33 ± 0.29 b	0.72 ± 0.01 a
Significance	ns	**	**	**	**	**

Means within each column followed by different letters are significantly different (*p* ≤ 0.05) according to Duncan’s multiple range test. ns, ** Non-significant or significant at *p* ≤ 0.01, respectively. All data are expressed as mean ± standard error, n = 3.

**Table 3:** Effects of anti-insect nets on dry matter (DM), pH, total soluble solids (TSS) content, hydrophilic antioxidant activity (HAA), ABTS antioxidant activity (ABTS AA), total phenols (expressed in dry weight, dw) and fruit total ascorbic acid (TAA; expressed in fresh weight, fw) of zucchini squash.

Treatments	DM	pH	TSS	HAA	ABTS AA	Total Phenols	TAA
	(%)		(°Brix)	(mmol Ascorbic ac. eq. 100 g^−1^ dw)	(mmol Trolox 100 g^−1^ dw)	(mg Gallic ac. eq. 100 g^−1^ dw)	(mg Ascorbic ac. 100 g^−1^ fw)
No-net	4.06 ± 0.17 b	6.31 ± 0.03	2.88 ± 0.09 b	9.93 ± 0.05 b	17.43 ± 0.66 b	165.96 ± 4.74 b	17.54 ± 0.29 b
50 mesh	4.84 ± 0.27 a	6.42 ± 0.03	4.26 ± 0.06 a	10.50 ± 0.12 a	22.02 ± 0.07 a	170.98 ± 3.43 b	19.46 ± 0.13 a
50 mesh AirPlus	4.88 ± 0.01 a	6.32 ± 0.05	3.08 ± 0.01 b	10.58 ± 0.14 a	23.10 ± 1.32 a	197.31 ± 2.45 a	19.01 ± 0.61 a
Significance	*	ns	***	*	**	**	*

Means within each column followed by different letters are significantly different (*p* ≤ 0.05) according to Duncan’s multiple range test. ns, *, **, *** Non-significant or significant at *p* ≤ 0.05, 0.01 and 0.001, respectively. All data are expressed as mean ± standard error, n = 3.

## Supplementary Materials

The following are available online at https://www.mdpi.com/2223-7747/9/10/1264/s1, Figure S1: Mean temperatures registered from April to July (2015–2018) by the meteorological station of Battipaglia (Salerno, Italy).
