# Supplementary material for: Improved Porosity of Insect Proof Screens Enhances Quality Aspects of Zucchini Squash without Compromising the Yield
Source: Plants (Basel). 2020 Sep 24;9(10):1264. doi: 10.3390/plants9101264 (PMC7600595; doi:10.3390/plants9101264)
Supplement: Supplementary file 1 [file plants-09-01264-s001.pdf]

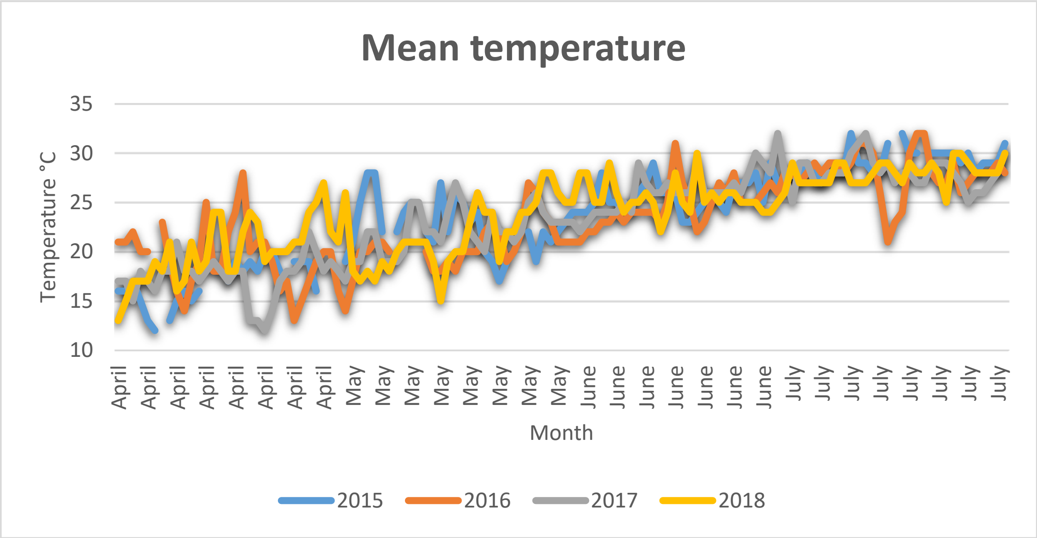

Supplementary figure S1: Mean temperatures registered from April to July (2015-2018) by the meteorological station of Battipaglia (Salerno, Italy).
